# Supplementary material for: Activation of regulatory T cells triggers specific changes in glycosylation associated with Siglec-1-dependent inflammatory responses
Source: Wellcome Open Res. 2021 Jun 1;6:134. [Version 1] doi: 10.12688/wellcomeopenres.16834.1 (PMC8844539; doi:10.12688/wellcomeopenres.16834.1)
Supplement: Peer review history

# Peer review: Reviewer response for version 1

**DOI:** 10.21956/wellcomeopenres.18569.r48238

**Published:** 2022-02-14

**Authors:** Hongshan Wei

**Affiliations:** 1 Beijing Ditan Hospital, Capital Medical University, Beijing, China

In the present manuscript, “Activation of regulatory T cells triggers specific changes in glycosylation associated with Siglec-1-dependent inflammatory responses”, the authors systematically observed the glycosylation changes after Tregs activation induced by Siglec‑1 binding. To the best of my knowledge, this was a first work focus on glycosylation changes in activated Tregs, especially the changes of sialylation. Using glycomic, RNA-Seq, and quantitative proteomic analysis, Wu *et al.* firstly found that expression of multiple glycogenes were involved in Tregs activation and Siglec-1 upregulation. In this process, proteomics analysis demonstrated that 49 Siglec-1 counter-receptors related with Tregs regulation. For further elucidating the role of these counter-receptors in Tregs regulation, the authors also observed the expression and glycosylation of Siglec-1 with Western blot and cytometry analysis. The authors assume that glycosylation and glycoform changes in activated Tregs resulted in increasing bounding activity of Siglec-1, and suppression of Treg expansion.

 This innovative study was a rigorous design, and with appropriate experimental methods. The writing is clearly expressed with appropriate logical level. Minor revision may be helpful for better understanding the logical conclusion.

1. As one of the important results, a global change of sialylation in activated Tregs was not observed, although the Siglec-1 binding activity was significantly increased. This result is different from Naito-Matsui (2014) and Redelinghuys (2011) previous reports. Several methods were used to confirm this result, such as glycomic analysis (Fig.4,5), Lectin and antibody-labelling analysis (Fig.3). Since the difference between its results and provious reports was resulted in Tregs and effector T cells, the CD25 and CD69 should accordingly be evaluated, as Kidder *et al.* (2013 ^1^) recently report.
2. As Villanueva-Cabello *et al.* (2019 ^2^) recently reported, not only were α2,3 and α2,6 linked sialic acids involved in Tregs activation, α2,8(polysialic acid) also complicated in this process. The 12E3-binding activity may be also evaluated, since the polysialic acid may be play an important role in orchestrating the CD4+T cells response.
3. As a typical study in glycobiological nature, multi-lectin chips may be further illustrating the characteristic of the Tregs sialylation, before and after activation.

Is the work clearly and accurately presented and does it cite the current literature?

Yes

If applicable, is the statistical analysis and its interpretation appropriate?

Yes

Are all the source data underlying the results available to ensure full reproducibility?

Yes

Is the study design appropriate and is the work technically sound?

Yes

Are the conclusions drawn adequately supported by the results?

Yes

Are sufficient details of methods and analysis provided to allow replication by others?

Yes

Reviewer Expertise:

protein glycosylation and liver disease

I confirm that I have read this submission and believe that I have an appropriate level of expertise to confirm that it is of an acceptable scientific standard.

## References

[1] : Sialoadhesin ligand expression identifies a subset of CD4+Foxp3- T cells with a distinct activation and glycosylation profile. *J Immunol* .2013;190(6) : 10.4049/jimmunol.1201172 2593-602 10.4049/jimmunol.1201172 23408841PMC3586974

[2] : Polysialic acid is expressed in human naïve CD4+ T cells and is involved in modulating activation. *Glycobiology* .2019;29(7) : 10.1093/glycob/cwz032 557-564 10.1093/glycob/cwz032 30989215

# Peer review: Reviewer response for version 1

**DOI:** 10.21956/wellcomeopenres.18569.r46375

**Published:** 2021-11-08

**Authors:** Karina Mariño

**Affiliations:** 1 Laboratory of Functional and Molecular Glycomics, Institute of Biology and Experimental Medicine, National Council for Scientific and Technical Research (IBYME-CONICET), Buenos Aires, Argentina

In this manuscript entitled “Activation of regulatory T cells triggers specific changes in glycosylation associated with Siglec-1-dependent inflammatory responses”, Wu *et al.* provide a comprehensive analysis of glycan changes that occur in activated iTregs, and its functional consequences with regards to interactions with the macrophage lectin, Siglec-1. Using in vitro-induced T reg cells (iTregs) as a model, the authors delve into the molecular basis for Siglec-1 binding to these cells and investigate the global and specific factors involved using a broad scope of methodologies (RNA-Seq, glycomics, and staining with lectins and anti-glycan antibodies). Moreover, they identify the counter-receptors for this lectin, which include a wide range of glycoproteins including CD43 (which was proposed previously to function as a counter-receptor for Siglec-1) but also others (i.e PD-1, adhesion molecules like CD166 and integrins). This was done by applying quantitative proteomics combined with proximity labeling, a methodology previously optimized by the authors in Siglec-1-erythrocyte interactions. 

 The manuscript is clear, very well written and the results are solid, novel and interesting. Moreover, they are obtained by robust methodologies and discussed and interpreted in a very appropriate manner. The article is scientifically valid in its current form. The experimental design, including controls and methods, is adequate; results are presented accurately and the conclusions are justified and supported by the data. The revisions I will suggest are minor in nature, but I consider them as relevant for a better understanding of the results provided.

 Further beyond the specific focus on Siglec-1 interactions with the iTreg glycome, and before going into the specifics of this work, this manuscript is highly valuable because it highlights the complexity of characterizing lectin-glycan interactions at a cellular level and exposes the need for complementary data based on different methodologies. 

 Going specifically to the comments regarding this work:

- All experiments are performed with iTregs obtained in vitro. In all the manuscript the cells are described as Tregs, not iTregs, and I think this should be clarified. 
- In Figure 4, they show that N-glycans showed an overall similar glycosylation and sialylation profile when resting and activated iTregs were compared, with very particular changes (a decrease in a bi-antennary glycan with core fucose and two NeuGc sialic acids, and a minor increase in m/z 2809 and m/z 2839, two mono-sialylated core fucosylated biantennary glycans decorated with alpha-gal). 
  The authors specifically show in this figure the changes in biantennary N-glycans. I understand they have analyzed the complete profile and only detected these few changes shown, with no alteration in other species (i.e. triantennary, tetrantennary)? Maybe this should be clarified in the legend or text. Extended data for N-glycomics relative to Figure 4 shows data for glycolipid analysis.
- Considering the different types of sialic acid linkage, have the authors analyzed the type of linkage in sialylated N-glycans (i.e. by treating with neuraminidases previous to the N-glycomics analysis)? If these experiments were not performed, then maybe the authors can briefly discuss potential differences in sialic acid linkages in the manuscript (which, for example, could reflect the decrease in St6Gal1 observed by RNAseq?).
- When performing RNA-Seq analysis, the authors assembled a specific list of 263 genes including glycosyltransferases, glycosidases, enzymes involved in amino sugar and nucleotide sugar metabolism, and sugar transporters, presented in Extended data Table 1, with corresponding results from average normalized count. 

In that sense;

- Have the authors analyzed the complete gene expression data by methods such as GSEA, to see if glycosylation pathways appear affected? As there is no mention of this, I assume the result was negative (this is a common issue with general analysis, as by analyzing all genes the differential expression of glycogenes is generally overviewed), but it would be really interesting as to compare results from the general gene pool with the changes they found when analyzing their specific list of genes. 
- Regarding those selected genes, could the authors provide a description on how those genes were selected? Taking into consideration databases ( i.e. Kyoto Encyclopedia of Genes and Genomes, Carbohydrate Active Enzymes, the Transport Classification Database)? Have they complied information form literature (i.e. Nairn *et al., *2008 ^1^). In this selected list of genes I could not find SLC17A5, a lysosomal sialic acid transporter.
- Extended Table 1: thinking about the non-specialized reader, it would be really useful if the authors could provide a schematic view of the expression of glycan-related genes by category. In that sense, they could present a) the table with the complete list of selected genes and a color code (or any type of division) showing their function (something similar to the supplementary table presented in Nairn *et al.*, 2008 ^1^), and maybe even highlighting in each section those relevant to the sialylation process; and b) the results in a separate heatmap.
- Regarding the p value, I would like to know if the values described in the Table correspond to pval or padj? Could the authors provide both?

Figure 9, extended data:

 Legend: “Mapping RNA-Seq data to the N-glycosylation pathway. Glycosylation related genes which had normalized counts above 100 in activated cells and had significant log2 fold changes (p<0.05) were mapped to the KEGG pathway using Pathview. For the pathway node which corresponds to multiple genes, the gene which had the biggest change was used for the mapping and the change of individual genes were then manually listed. A) Mapping to N-glycosylation pathway. The genes involved in synthesizing and transferring the N-glycan precursor to glycosylation sites increased upon Treg activation. B) Manually listing of genes mapped to the OST node. Four of the 5 components in OST enzyme complex were increased in activated Tregs.”

 To the best of my understanding, OST has more than five components. Are the authors only showing those with significant dysregulation? 

 Finally, I think it would be useful to change the legend for panel B from “Manually listing of genes mapped to the OST node” to “Dysregulation of genes mapped to the OST node”

 Figure 10, extended data: 

 “B) Manual listing of genes that mapped to the node 2.4.1.41. Galnt3 was the only one which showed increased expression upon Treg activation.”

 I would suggest changing this legend to: 

 “Dysregulation of genes mapped to the node 2.4.1.41. Galnt3 was the only one which showed increased expression upon iTreg activation”

- Figure 7: maybe the authors could present the data in a heatmap? I believe that this change in the presentation of data could really benefit the manuscript.

Minor comment regarding Figure 7, Legend:

 It reads:

 “Normalized mRNA counts for genes involved in sialylation. Resting and activated Tregs from 4 biological replicates were analzyed by RNA-Seq. Genes involved in synthesis of (A) α2,3-linked sialic acid, (B) α2,6-linked sialic acid, (C) α2,8-linked sialic acid and (D) modification or removal of sialic acid are listed”.

 It should read: 

 “Normalized mRNA counts for genes involved in sialylation. Resting and activated Tregs from 4 biological replicates were analzyed by RNA-Seq. Genes involved in synthesis of (A) α2,3-linked sialic acid, (B) α2,6-linked sialic acid, (C) α2,8-linked sialic acid and (D) modification or removal of sialic acid are shown.”

 Lastly, and after looking at the results, maybe the authors can consider changing the title of this section of the manuscript? Currently it states “RNAseq did not reveal specific changes predicted to have major effects on Siglec-1 recognition following Treg activation”, but the results show dysregulation of several enzymes related to sialylation and glycosylation. The authors later on discuss the effects of this dysregulation very well, postulating that it may affect specific glycoconjugates. 

 By quantitative proteomics combined with proximity labelling, they identify a number of counter-receptors for Siglec-1 in iTregs, comprising approximately 5% of the total membrane proteins. They also show that most counter-receptors are heavily glycosylated and that glycosylation density is an important determinant of functioning as a Siglec-1 counter-receptors. 

 Lastly, they show that independently of upregulation after activation, CD48 and PD-1 show increased glycosylation (Figure 11C,D). For PD-1, this was confirmed following treatment of affinity-purified PD-1 with PNGase F and sialidase.

 Can the authors identify the sialidase used in these experiments? I could not find it in materials and methods. 

 Lastly, they conclude that mechanisms mediating the increase of Siglec-1 binding in activated iTregs could involve complex protein- and lipid-dependent modification of sialylation at an individual carrier level. I believe this work is very valuable, considerably increases knowledge on Siglec-1-iTregs interactions, and points at the impact of Siglec-1 on PD-1 function in T cells as a very interesting area.

Is the work clearly and accurately presented and does it cite the current literature?

Yes

If applicable, is the statistical analysis and its interpretation appropriate?

Yes

Are all the source data underlying the results available to ensure full reproducibility?

Yes

Is the study design appropriate and is the work technically sound?

Yes

Are the conclusions drawn adequately supported by the results?

Yes

Are sufficient details of methods and analysis provided to allow replication by others?

Yes

Reviewer Expertise:

Glycobiology, Glycomics, lectin-glycan interactions

I confirm that I have read this submission and believe that I have an appropriate level of expertise to confirm that it is of an acceptable scientific standard.

## References

[1] : Regulation of glycan structures in animal tissues: transcript profiling of glycan-related genes. *J Biol Chem* .2008;283(25) : 10.1074/jbc.M801964200 17298-313 10.1074/jbc.M801964200 18411279PMC2427342
